# DvD: An R/Cytoscape pipeline for drug repurposing using public repositories of gene expression data

**DOI:** 10.1093/bioinformatics/bts656

**Published:** 2012-11-04

**Authors:** Clare Pacini, Francesco Iorio, Emanuel Gonçalves, Murat Iskar, Thomas Klabunde, Peer Bork, Julio Saez-Rodriguez

**Affiliations:** ^1^European Bioinformatics Institute (EMBL-EBI), Wellcome Trust Genome Campus, Cambridge, UK CB10 1SD, ^2^Wellcome Trust Sanger Institute, Wellcome Trust Genome Campus, Cambridge, UK, CB10 1HH, ^3^Structural and Computational Biology Unit, European Molecular Biology Laboratory, Meyerhofstrasse 1, 69117 Heidelberg, Germany and ^4^Sanofi-Aventis Deutschland GmbH, Frankurt am Main, Germany

## Abstract

**Summary:** Drug versus Disease (DvD) provides a pipeline, available through R or Cytoscape, for the comparison of drug and disease gene expression profiles from public microarray repositories. Negatively correlated profiles can be used to generate hypotheses of drug-repurposing, whereas positively correlated profiles may be used to infer side effects of drugs. DvD allows users to compare drug and disease signatures with dynamic access to databases Array Express, Gene Expression Omnibus and data from the Connectivity Map.

**Availability and implementation:** R package (submitted to Bioconductor) under GPL 3 and Cytoscape plug-in freely available for download at www.ebi.ac.uk/saezrodriguez/DVD/.

**Contact:** saezrodriguez@ebi.ac.uk

**Supplementary information:** Supplementary data are available at *Bioinformatics* online.

## 1 INTRODUCTION

Multiple methods based on matching gene expression signatures have been proposed to identify anti-correlated drug and disease profiles (Hu *et al.*, 2009; Sirota *et al.*, 2011). The central paradigm is that a drug compound, which shows the opposite effect on gene expression to the observed for a disease could be used to treat that particular disease. These methods have resulted in hypotheses of differential uses (repurposing) for existing compounds that have been validated experimentally. This highlights the potential power of mining existing safe compounds for repurposing, which does not require the expensive and extensive initial design and clinical phases of drug discovery. It is expected that analysing new and existing data from public repositories such as Array Express (www.ebi.ac.uk/arrayexpress/), Gene Expression Omnibus (GEO) (www.ncbi.nlm.nih.gov/geo/) and the Connectivity Map (CMap) (www.broadinstitute.org/cmap/) using these methods will become increasingly popular in computational drug discovery (Iorio *et al.*, 2012).

Motivated by this, we have developed Drug versus Disease (DvD), an R package to ‘match’ drug and disease profiles. This is done by making use of gene set enrichment analysis (Subramanian *et al.*, 2005) and visualizing the final results in networks containing clusters of similar drugs and diseases. DvD differs from existing web servers and databases (such as ProfileChaser, MARQ and SPIED, see Supplementary Material) in that it can dynamically access both Array Express and GEO to generate input profiles. With DvD, users can automatically compare input profiles to reference drug data from CMap and disease profiles curated from GEO. This reference data has associated networks where, unlike similar tools, drugs or diseases exerting similar effects on transcription are grouped into clusters. DvD is flexible, offering customizable and default data options for the input profiles and the reference data. The Cytoscape (www.cytoscape.org/) plug-in provides a user interface to the full DvD pipeline, as well as a visualization platform for the results. In the final networks, drug or disease nodes are linked to the DrugBank (www.drugbank.ca/) and Medical Subject Headings (MeSH) (www.ncbi.nlm.nih.gov/mesh/) web browsers, respectively.

## 2 ANALYSIS PIPELINE

**Fig. 1.**
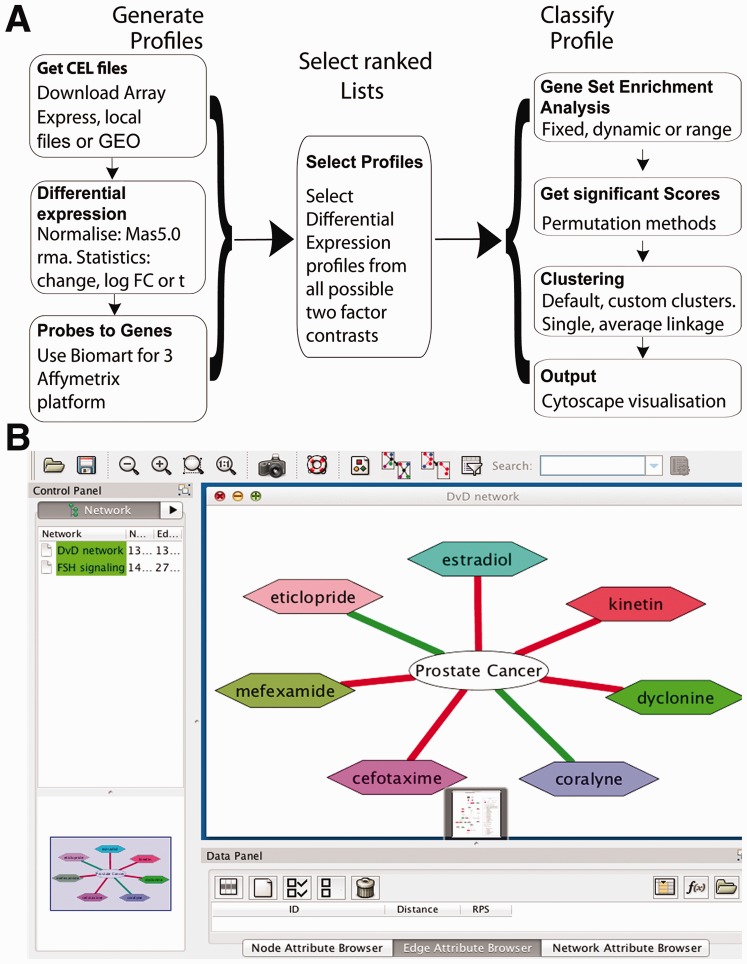
DvD pipeline. (**A**) *GenerateProfiles* and *ClassifyProfile* are wrapper functions whose stages are shown in the vertical flow charts. *GenerateProfiles* imports the data and normalizes CEL files where necessary. *Probes to Genes* maps Affymetrix probes to HUGO gene symbols using BiomaRt. Finally, differential expression statistics are calculated using limma. *SelectRankedLists* can be used to select a subset of the contrasts output from generate profiles. *Classifyprofile* can take input from *generateProfiles*, *selectrankedlists* or the users own preprocessed data. This function calculates and identifies significant Enrichment scores and produces corresponding network files. (**B**) Example visualization produced by the Cytoscape plug-in for the prostate cancer profile (gse17906). Red edges are for inverse correlations and green positive

The DvD pipeline provides a number of processing options to generate genome-wide expression profiles from microarray experiments (see Fig. 1A). Options to import data from local directories and Array Express or GEO are supported (Davis *et al.*, 2007; Kauffmann *et al.*, 2009). Data are normalized using either rma or mas5 (Irizarry *et al.*, 2003). DvD will automatically annotate, filter and combine probes to HUGO genes for the Affymetrix platforms HG-U133A, HG-U133A-2 and HG-U133-Plus2 using BiomaRt (Durinck *et al.*, 2009). Annotation files can be passed to DvD to process data from other platforms. Probes mapping to multiple gene identifiers are removed. Multiple probes mapping to the same gene can be converted using the average or maximal intensities, median polish or by selecting the probe with the highest variance across all arrays.

DvD expects as input either a drug or disease profile. Using this, and the experimental design factors from the Array Express and GEO databases, DvD identifies a main factor for the experiment. In this way, unlike existing methods, DvD is able to calculate differential expression (Smyth *et al.*, 2004) between levels of the main factor, stratified by a second factor (see Supplementary Material). Given a ranked list of differential expression, either from DvD or by providing preprocessed data, DvD defines gene sets. These can be a fixed number chosen *a priori* or determined by the number of significantly differentially expressed genes. Enrichment scores are then calculated using either the Kolomgorov–Smirnov-based statistic (Iorio *et al.*, 2010; Subramanian *et al.*, 2005) or the weighted signed statistic (Zhang *et al.*, 2008) by querying the reference dataset with these gene sets. Significance of enrichment scores is determined by comparison with an empirical null distribution. Scores can be corrected for multiple hypothesis testing using either Benjamini–Hochberg correction or *q*-value method. Profiles producing significant scores are finally assigned to clusters using single or average linkage. In the latter case, the average score for a cluster is defined as either the mean or the median distance to each profile in the cluster.

### 2.1 DvD data packages

Two associated data packages, cMap2data and DrugVsDiseasedata provide default reference ranked expression profiles and clusters. The cMap2data is based on the CMap version 2 dataset, which contains 6100 hybridizations of 1309 different compounds. The merged profiles obtained by Iorio *et al.* (2010) were used to generate a single gene level ranked profile for each of the 1309 compounds. Disease profiles are defined for 45 diseases based on data from GEO with associated clusters. Analysis of the disease and compound clusters demonstrated viable results. For example, one drug cluster was significantly enriched for Histone deacetylase inhibitors, and a disease cluster was found that contained multiple profiles of different cancers (see Supplementary Material).

### 2.2 Cytoscape plug-in

The Cytoscape plug-in uses the Rserve framework. The main R wrapper provides a graphical interface to the full DvD pipeline and information on the drug and disease profiles contained in the DvDdata package (Fig. 1B). This is obtained from DrugBank for the drugs and MeSH for the disease profiles. Furthermore, it links DvD to other Cytoscape plugins for further analysis. This could include mapping differential expression profiles associated with drug candidates obtained from DvD on to signalling networks (see Supplementary Material).

## 3 RESULTS

To illustrate the use of DvD in drug repurposing we analysed several disease datasets available from GEO. We compared them with the 1309 compounds in the CMap using DvD and considered significant those connections with *q*-value < 0.05. A prostate cancer profile (gse17906) had seven significant matches with five being negative (Fig. 1B). The strongest negative correlation was with Estradiol, a known treatment for prostate cancer. For a breast cancer profile (gse5847), we found the third highest inverse correlation with Tamoxifen, a similarly well-known treatment for breast cancer. The compound scoring highest was Ranitidine, a Histamine receptor type-2 (H2) antagonist, which has previously analysed as a potential treatment for breast cancer (Bolton *et al.*, 2000). Finally, we analysed a type II diabetes profile (gse15653) and found Phenformin and Torasemide at the seventh and ninth highest therapeutic scores, respectively. Interestingly, Finasteride scored higher than both of these known treatments for type II diabetes, with the third strongest negative correlation. Finasteride inhibits the type-2 5 alpha-reductase enzyme, which converts testosterone to dihydrotestosterone. Testosterone is known to be important in glucose homeostasis and lipid metabolism (Saad, 2009). These results were not obtained when analysing these three profiles using CMap (Supplementary Material), showing the value in combining replicate experiments to generate profiles for comparison.
